# The Relations of Science Task Values, Self-Concept of Ability, and STEM Aspirations Among Finnish Students From First to Second Grade

**DOI:** 10.3389/fpsyg.2019.01449

**Published:** 2019-07-02

**Authors:** Janica Vinni-Laakso, Jiesi Guo, Kalle Juuti, Anni Loukomies, Jari Lavonen, Katariina Salmela-Aro

**Affiliations:** ^1^Faculty of Educational Sciences, University of Helsinki, Helsinki, Finland; ^2^Institute for Positive Psychology and Education, Australian Catholic University, North Sydney, NSW, Australia; ^3^Viikki Teacher Training School, Faculty of Educational Sciences, University of Helsinki, Helsinki, Finland; ^4^Department of Childhood Education and Centre for Education Practice Research, University of Johannesburg, Soweto, South Africa; ^5^Collegium Helveticum – ETH Zürich, Zurich, Switzerland

**Keywords:** expectancy-value theory, intrinsic value, cost, self-concept of ability, STEM occupational aspirations, gender differences, elementary students

## Abstract

According to modern expectancy-value theory, students’ motivation in school subjects begins to vary at the very beginning of their school careers, showing a task-specific pattern of motivation. However, there is no clear evidence in the literature on how students’ value beliefs are formed and interact with each other in early elementary schools. Using the longitudinal structural equation modeling, this study examined relations between science-related task values (i.e., intrinsic value and cost), self-concept of ability, and future occupational aspirations based on first graders and 1-year follow-up from seven schools in Helsinki (*N* = 332; ages = 7 and 8 years; girls = 51%). Results showed that the students who had a high science-related self-concept of ability and intrinsic value tended to perceive low cost of science learning. Science-related self-concept of ability was the most stable construct, while in intrinsic value and cost, there were significant levels of fluctuation across the first and second grades. A high science-related self-concept of ability in the first grade predicted a lower cost value in the second grade, and a high science-related intrinsic value was a marginally significant predictor of future occupational aspirations in science, technology, engineering, and mathematics (STEM). Mean-level differences revealed that the girls’ science-related self-concept of ability, intrinsic value, and cost remained the same in both grades, while the boys’ self-concept of ability decreased. The girls’ mean levels in science-related intrinsic value were higher than those of the boys, while students’ self-concept of ability and cost were similar across gender in both grades. A cross-lagged panel model revealed that the girls reported more STEM occupational aspirations than the boys in the second grade, while controlling for the motivational beliefs. In summary, the results indicate that a high-level of science interest in young students predicts STEM occupational aspirations; high girls’ intrinsic value in early science education does not steer them away from STEM occupations; boys’ task motivation might be at greater risk of decline during early science education.

## Introduction

During the last decade, increased attention has been given to low student interest and engagement in science learning and science-related careers. For example, Horizon 2020, a European Union Research and Innovation program, emphasized the need for a science education supporting students in developing positive attitudes toward science and nurturing their curiosity and cognitive resources (Ryan, 2015). Research on students’ science motivation and adolescents’ educational and occupational aspirations has also increased in recent years [see Tytler (2014) for a review]. Trends consistently show a decrease over time in students’ positive attitudes and motivation toward science and their pursuit of science-related careers. Recent international large-scale assessments, such as the Programme for International Student Assessment (PISA) and the Trends in International Mathematics and Science Study (TIMMS), have showed that Finnish students’ science-related achievement and motivation have been declining (Martin et al., 2016; OECD, 2016). According to the PISA 2015 report (OECD, 2016), Finnish ninth graders’ interest in and enjoyment of science, technology, engineering, and mathematics (STEM) are below the average for OECD countries, and their occupational expectations in the STEM field are among the lowest in OECD countries (OECD, 2016).

Gendered pathways into STEM have also received growing attention in the literature [see Watt (2016) for a review]. Women are clearly underrepresented in math-intensive science education and STEM careers (OECD, 2015), stimulating research into possible explanations. Some findings suggest that stereotypes might play a role in students’ future occupational aspirations and that women have not stereotypically been seen as scientists (Chambers et al., 2018; Miller et al., 2018). Female students’ self-concept of ability in science is lower than that of male students, despite the fact that girls often outperform boys in school science (Watt, 2016). There are also gender differences in attitudes to science among Finnish students, with boys showing higher enjoyment and girls holding higher instrumental value in science learning (OECD, 2016). Still, the gender gap favoring boys in bachelor’s degrees obtained in science remains significant in Finland (OECD, 2015). High academic motivation in science has been positively linked to deeper engagement, persistent learning, better knowledge acquisition, and higher aspirations in that domain, which prepares individuals to pursue further education and careers in STEM fields [see Wang and Degol (2013) for a review].

Until now, studies of science motivation and STEM aspirations and their development have mainly focused on middle and high school students. In order to form a more comprehensive picture of the phenomenon, and to understand middle and high school students’ science-related motivation and aspirations regarding STEM, we need to examine the point at which decline in students’ science motivation begins to emerge, how stable their motivational beliefs are, and what factors relate to this development. Thus, in order to investigate the trajectories of STEM motivation from the beginning of elementary students’ school careers, we need to address their science-related motivation. In addition, most previous studies of young students’ STEM motivation have been conducted in the United States; therefore, more research is needed in other educational contexts.

### Task Motivation in Early Science Learning

In this study, we draw on the modern expectancy-value theory (EVT) framework (Eccles, 2009). EVT (Eccles et al., 1983; Eccles, 2009) posits that achievement-related performance and choices are most directly influenced by students’ expectations of success on achievement-related tasks and their subjective assessments of the relative value of different achievement-related tasks. Eccles’ EVT of achievement-related choices is a major theoretical framework for studying achievement motivation. It has been widely used to tackle both individual and gender differences in educational and career choices [see Wang and Degol (2013, 2017) and Watt (2016) for reviews]. Within the academic domain, Eccles et al. (1983) operationally defined *expectancies of success* as children’s beliefs of how well they will do on an upcoming task. Expectancies of success are children’s evaluations of their current abilities as well as how they think they compare to other students (Wigfield et al., 2016) in a given task. Thus, we use the term task-specific self-concept of ability. Wigfield and Eccles (1992) and Eccles and Wigfield (2002) also distinguished between multiple components of subjective task values: *intrinsic value* (enjoyment or liking), *utility value* (the usefulness of a task for helping to fulfill personal goals), *attainment value* (relevance of a task to one’s sense of self, identity, and core personal values), and *costs* (perceived negative aspects of making a specific choice).

*Intrinsic value* refers to the extent to which an individual gains enjoyment from performing an activity (Eccles, 2009, 2011). *Cost* refers to the things that students perceive they are investing or giving up in order to engage in a task (Flake et al., 2015), including the degree of potential loss of time; effort demands; the loss of valued alternatives, such as spending time with friends; and additional negative experiences, such as stress. According to EVT (Eccles et al., 1983; Eccles, 2009), people are most likely to select those tasks for which they hold the highest expectations for success and the highest levels of subjective task value.

Students’ achievement-related beliefs and attitudes play an important role in academic environments by directing their behavior and effort in learning situations (Eccles, 2009; Marsh and Martin, 2011). Students who have a positive self-concept of ability and intrinsic value in specific academic subjects are likely to perform better and be more engaged in school than those who have a less positive self-concept of ability in a given subject. Previous studies have shown that students hold high beliefs in their abilities at the beginning of elementary school and that they are highly optimistic about their competences in different areas and domains (Stipek and Mac Iver, 1989; Wigfield et al., 2016). The trajectories of students’ self-concept of ability decline from the elementary to the middle and high school years, although some domain-specific features can be identified in these emerging trajectories (Jacobs et al., 2002). Studies have provided several reasons for the decline in students’ self-concept of ability, including children’s developmental changes, increasing social comparisons among students, and environmental changes in the school context, for example, increasing numbers of subjects and teachers and greater emphasis on grades (Stipek and Mac Iver, 1989; Marsh et al., 1995; Harter, 2012).

Intrinsic motivation is well known to be associated with ability beliefs, i.e., those with a higher self-concept of ability are more willing to engage in learning processes and enjoy learning. Thus, it is no wonder that, upon entering school, elementary students have high academic interest (e.g., Viljaranta et al., 2016). However, this interest soon starts to decline across domains (Wigfield et al., 1997; Gottfried et al., 2001; Jacobs et al., 2002). Furthermore, previous EVT studies have tended to show moderate to large correlations between academic self-concept of ability and the value components [see Wigfield and Eccles (2002) and Wigfield et al. (2009, 2016) for reviews]. Self-concept of ability is more highly correlated with intrinsic value than with the other value components within a specific domain (Wigfield et al., 2009, 2016), and the positive correlation between task motivation and self-concept of ability has been found to strengthen with age (Wigfield et al., 1997; Fredricks and Eccles, 2002; Jacobs et al., 2002).

A general downward shift in students’ interest from elementary to middle school is also evident in the science domain (Gottfried et al., 2001). In general, academic interest is rather stable, and this stability increases with age across subject domains (Gottfried et al., 2001). This kind of development poses a challenge for children who begin their school careers with low science motivation. The trend of declining motivation affects students’ achievement and increases the risk of dropout from science education later on and, as a consequence, from STEM careers.

In addition to low interest, students might also experience costs that affect their motivation as a negative valence of a task (Barron and Hulleman, 2015). Cost is the least studied component of EVT, and it is distinct from other components of the EVT model (see Flake et al., 2015). In addition, students have been found to report different types of cost, some of which include the requirement of too much effort, emotional/psychological demands, and loss of other valuable opportunities. It has been shown that perceived cost can detract students from engaging in a task or activity and that it can be a powerful predictor of career and education-related outcomes in middle school, high school, and college students (e.g., Battle and Wigfield, 2003; Wigfield and Cambria, 2010; Perez et al., 2014; Flake et al., 2015; Jiang et al., 2018). However, research on elementary students’ EVT motivation including perceived cost is lacking.

To sum up, in general, self-concept of ability and intrinsic motivation decrease as students proceed from the first grade onward. Alongside this development, interest may play a role in the formation of self-concept of ability. However, little is known about elementary students’ experience of cost, particularly in the context of science. First graders would probably engage in a task that is interesting and fun rather than a task that they evaluate as useful for their future or important for their personal selves (Wigfield and Eccles, 1992; Eccles and Wigfield, 2001). In a similar vein, students would most probably disengage from tasks they perceive as overly demanding or emotionally exhausting. In this study, our primary aim is to explore the stability and associations among first graders’ science-related self-concept of ability, intrinsic value, and perceived cost during a 1-year follow-up.

### Task Motivation and Academic Aspirations

Research based on EVT has demonstrated that self-concept of ability and value beliefs represent the most proximal precursors of academic achievement, effort, school engagement, and educational aspirations (e.g., Marsh et al., 1995; Eccles, 2009; Watt et al., 2012; Wang et al., 2013; Guo et al., 2015a). Several studies showed that interest in a certain subject domain is related to academic achievement in that domain (e.g., Harackiewicz and Hulleman, 2010; Guo et al., 2015b, 2017). Positive associations of intrinsic value and self-concept of ability with achievement have also been found among elementary school students (Denissen et al., 2007; Viljaranta et al., 2014) and in the context of science (Guo et al., 2018b). According to EVT, the cost component of task values is assumed to dampen students’ motivation, and it is strongly and negatively related to expectancy and moderately and negatively related to value, long-term interest, course grades, and overall motivation (Flake et al., 2015). Thus, it is important to differentiate and consider cost, self-concept of ability, and intrinsic value among elementary school students to further disentangle the relationships between self-concept of ability and positive and negative value beliefs in achievement-related outcomes of future occupational aspirations.

The recent study on elementary students’ career aspirations demonstrates that children’s aspirations are shaped from a young age (Chambers et al., 2018). Students’ attitudes shape their interests and later behavior. Thus, student motivation determines the choices students make about their educational pathways. Highly motivated students are more likely to choose courses and aspire to careers that correspond with the subjects in which they are motivated (Simpkins et al., 2006; Chow et al., 2012; Wang, 2012). Students’ intrinsic value and academic self-concept in mathematics and science have been found to predict their STEM aspirations in middle and high school (Wang and Degol, 2013; Guo et al., 2015b). Moreover, longitudinal tracking has showed that students who do not express STEM-related aspirations at the age of 10 years are unlikely to develop STEM aspirations by the age of 14 years, and consequently are less likely to pursue science subjects (Archer et al., 2013). In contrast, perceived cost is a negative predictor of interest and performance outcomes (Perez et al., 2014; Barron and Hulleman, 2015; Flake et al., 2015; Jiang et al., 2018). These findings further underline the importance of student motivation for long-term academic and career success. However, whether these motivational beliefs are related to elementary students’ future occupational aspirations has not been tested. In fact, we know very little about the factors that influence early career aspirations, despite the fundamental role of aspirations in individuals’ career choices and development throughout the lifespan.

Of particular relevance is that no previous study has integrated science-related self-concept of ability, intrinsic value, and cost to determine the extent to which these beliefs and emotions relate to future occupational aspirations among early elementary students. Thus, we have chosen to focus on self-concept of ability and positive and negative aspects of task value – namely, intrinsic value and cost – to examine the associations of these constructs among first and second graders in science learning, and how these constructs are related to students’ occupational aspirations.

### Gendered Science Motivation and Occupational Aspirations

Previous findings show that students’ self-concept of ability and intrinsic value become gendered, especially in relation to mathematics and literacy (see Eccles et al., 1993; Wigfield et al., 1997; Jacobs et al., 2002). Girls’ self-concept of ability in mathematics is found to be lower than that of boys, but girls show a lower decline over time (Fredricks and Eccles, 2002), indicating that the gender gap decreases over time. Meanwhile, boys’ self-concept of ability in language and arts is lower and declines more than that of girls (Jacobs et al., 2002). According to a recent meta-analysis (Miller et al., 2018), the last five decades have witnessed a developmental change in children’s gender-science stereotypes. In a draw-a-scientist study, children in the 1960s almost exclusively depicted scientists as males; in 2000, significantly more children depicted female scientists than their 1960s counterparts. In another study, Bian et al. (2017) observed that not only gender-science stereotypes were still prevalent, but they also started to emerge early. Bian et al. (2017) also found that children perceived males as more intellectual than females, which had a clear influence on their interests in selecting tasks that are described to be easy or difficult, even at the age of 6 years. However, no gender differences in science-related self-concept were found in preschool and early elementary school-aged children (Leibham et al., 2013).

Boys have been shown to hold higher interest in science in early education, although high science interest in preschool predicted higher self-concept and achievement for 8-year-old girls (Leibham et al., 2013). Particularly in the case of the early elementary school years, it appears that interest helps build a higher self-concept of ability. Various studies have found that, as early as elementary school, boys hold higher intrinsic values in mathematics, while girls hold higher intrinsic values in language (Eccles et al., 1983; Jacobs et al., 2002). These gendered value beliefs also feature among secondary school students (e.g., Gaspard et al., 2015).

As noted, cost is salient in student motivation and is linked to several educational outcomes (Flake et al., 2015). Watt (2016) investigated adolescents’ gender differences in science and found different types of cost to be differently gendered. For example, girls experienced greater psychological cost (e.g., “It frightens me that math/science courses are harder than other courses”), while boys experienced more social cost (e.g., “I’m concerned that working hard in math/science classes might mean that I lose some of my close friends”) in their science learning in Grade 10. In terms of effort-related cost, no gender differences were found. In this study, we are interested in studying whether the experience of cost emerges from the first school years and whether this experience is gendered. This might provide additional insights as to why girls and boys end up valuing different subjects and choosing different career paths, in spite of their equal competences.

Regardless of the predictive power of cost on educational outcomes, to our knowledge, no previous study has examined the cost component of elementary students’ science learning. Moreover, no study has examined the possible gendered patterns in science motivation at such an early age (for an exception, see Oppermann et al., 2018). It is crucial to examine these aspects of science motivation in early education in order to understand why students, especially girls, are opting out of science education and careers. In addition to our primary aim in the present study, we examine if there are gender differences in young students’ science-related motivational beliefs and aspirations.

## The Current Study

In this study, we draw on the framework of modern EVT (Eccles, 2009) to analyze a large sample of first-grade students (aged 7 years) in Finland, who were studied twice, 1 year apart. We examined science-related self-concept of ability, intrinsic value, and cost; the stability of these factors; and their unique contributions to science motivation development in students. In addition, the study investigates the extent to which science-related self-concept of ability, intrinsic value, and cost predict students’ future STEM occupational aspirations. Finally, we address gender differences in students’ self-concept of ability, task values, and STEM aspirations. Of central importance, the present study captures the positive and negative valence of science task values to explore the unique power of first graders’ science-related self-concept of ability and task motivation on their future STEM occupational aspirations in the second grade.

### Research Question 1: What Are the Autoregressive and Cross-Lagged Effects Between Science-Related Self-Concept of Ability, Intrinsic Value, and Cost Across the First and Second Grades?

Our first aim is to examine the mean-level stability and rank-order stability of science-related self-concept of ability, intrinsic value, and cost from Grades 1 to 2. Young children tend to be optimistic about their abilities across different academic subjects, and they place high subjective task values on different school subjects (Viljaranta et al., 2016). However, as they gather more experience with different academic subjects, gain more cognitive skills, and experience a wider range of school environments, such optimism changes to pronounced realism and even pessimism for many children (Stipek and Mac Iver, 1989; Wigfield et al., 2016). Based on these results, we expect first graders to have high self-concept of ability and intrinsic value beliefs at the beginning of their school career, and that their self-concept might decrease from Grade 1 to Grade 2. Moreover, based on prior literature on the development of task values, we assume that students’ motivational beliefs will not be very stable at the age of 7–8 years (Wigfield and Eccles, 1992; Eccles and Wigfield, 2001). In the science domain, cost has not been previously studied in students of this age cohort. Thus, we are unable to hypothesize the stability of perceived cost or whether first graders perceive science learning as exhausting and demanding.

We also aim to examine the cross-lagged relations of science-related self-concept of ability, intrinsic value, and cost across the first and second grades. In line with the literature (Eccles et al., 1993; Wigfield and Eccles, 2002; Wigfield et al., 2009), we expect self-concept of ability to be positively related to intrinsic value and cost to be negatively related to self-concept of ability and intrinsic value (Barron and Hulleman, 2015).

### Research Question 2: Do First Graders’ Science-Related Task Values Predict Their Future STEM Occupational Aspirations 1 Year Later?

In middle and high school, students’ science motivation has been found to predict educational and occupational aspirations (Wang and Degol, 2013; Guo et al., 2018a). Based on EVT, we hypothesize that students’ high intrinsic value and self-concept of ability in science are positively associated with their STEM aspirations and that perceived cost in science is negatively associated with STEM aspirations a year later.

### Research Question 3: Are There Gender Differences in Students’ Science-Related Task Values and STEM Occupational Aspirations in the First and Second Grades?

It has been shown that boys hold higher self-beliefs and intrinsic value in science in early education (Leibham et al., 2013). However, recent findings indicate differences in girls’ and boys’ value beliefs in the physical (e.g., physics) and life (e.g., biology) sciences, and that there are increasing gender differences in physics and biology in middle school (Gaspard et al., 2017; Guo et al., 2018b). In line with previous findings, we expect boys to have a higher self-concept of ability and intrinsic value toward science at the beginning of elementary school. Gender equality is strongly promoted in Finnish society and emphasized in school; however, despite these efforts, gendered trajectories persist in education and occupations. Therefore, we are unable to formulate a hypothesis about the effect of gender on students’ future STEM occupational aspirations.

## Materials and Methods

### Participants and Procedure

The study sample consisted of 332 students, who underwent two rounds of testing: in the first grade and, 1 year later, in the second grade (Time 1: median age = 7 years, *SD* = 0.319, 188 girls, 144 boys; Time 2: median age = 8 years, *SD* = 0.389, 188 girls, 144 boys). The data were collected in 2016 and 2017 during the spring semester. The students were from 7 schools and 20 classes (two to five classes per school) located in the eastern suburbs of Helsinki, characterized by mixed levels of socio-economic status. There were one to two researchers per class, instructing and guiding the data collection. First, the students were introduced to the principles of answering a questionnaire and what the scales meant. It was emphasized that the most important thing was to answer honestly, that each opinion was valuable, that the responses would not be used for classroom evaluation purposes, and that their teachers would not see the responses. The students answered the questionnaires as part of a guided activity; the researcher read each item aloud, explaining unfamiliar concepts as needed. Special emphasis was placed on explaining the reversed items and how the scale should be interpreted with respect to those items. Students who had difficulties with the Finnish language or with following these procedures were assisted. The data were collected at the beginning of the spring semester in the student’s first year of school to ensure that they had acquired basic reading skills and could more easily follow the questionnaire. At that time, they had half a year of experience studying science, or environmental studies, as it is called in the curriculum. Thus, it can be expected that the students were familiar with the context of the questions and understood the questions when they provided their answers. After the group completed each page of the questionnaire, they took a short break. The questionnaire was completed during one lesson (about 45 min).

The research project follows the strict national ethical guidelines of scientific studies of human subjects set by the Finnish Advisory Board of Research Integrity (TENK^1^), which are in line with the European Code of Conduct for Research Integrity of All European Academies (ALLEA) and the General Data Protection Regulation recently issued by the European Commission. The University of Helsinki Ethical Review Board in the Humanities and Social and Behavioural Sciences sanctions these national guidelines (TENK) and provides six descriptions of research designs that need to be handed for ethical reviews^2^. According to these guidelines, this study did not require ethical review, and therefore, no ethics application was made. Furthermore, to follow good scientific practice, the research plan was pre-examined and approved by the Education Division of the city of Helsinki. Since the participants of the study were elementary school-aged children, the study description and the permission forms for participation were sent to the students’ parents beforehand. Parental consent was sought, and parents’ were asked to either give or decline permission to take part in the study. Written active parental consent was obtained from all the student participants. Data collection was integrated in students’ normal classroom activities. The headmasters and teachers of the participating schools were informed about and agreed to the data collection schedule. The class teacher organized separate activities for those students who did not have permission to participate in the study.

### The Finnish Science Education Context

In Finland, students start school in the year they turn 7. Before school begins, children attend preschool for 1 year. The concept of science refers to school science, or environmental studies, as defined in the Finnish National Core Curriculum for Basic Education (NCCBE, 2014). According to the NCCBE (2014), environmental studies are an integrated subject, which comprises the knowledge fields of biology, geography, physics, chemistry, and health education. Its key objective is to guide students to understand the impact of the choices made by humans on life and the environment. The multidisciplinary nature of the subject requires that students learn to acquire, process, produce, present, evaluate, and appraise information in different situations (NCCBE, 2014). The viewpoints of scientific information and critical thinking are emphasized. In the first and second grades, the teaching and learning of environmental studies is structured into units in which the students’ own environment, the students themselves, and their actions are examined. The students’ curiosity and interest in phenomena in their surroundings are stimulated through problem-solving and inquiry assignments based on play. Students practice analyzing and naming elements in their surroundings and examine issues related to their own well-being and safety. The objectives of the subject in the first and second grades emphasize the development of environmental awareness, attitudes, values; developing research and working skills; and understanding the meanings of basic concepts, such as processes and structures in nature, the environment, and energy (NCCBE, 2014).

### Measures

#### Task Motivation in Science

Students’ science-related self-concept of ability, intrinsic value, and cost were examined using a task-value instrument based on EVT (Eccles et al., 1983; Eccles, 2009). The scale included self-concept of ability in science (i.e., “I am good at science,” “I am good at schoolwork on this subject,” and “Schoolwork on this subject is easy for me”; Time 1 α = 0.66, Time 2 α = 0.63), science intrinsic value (i.e., “I find science fun,” “I like to do schoolwork on this subject,” and “I just like this subject”; Time 1 α = 0.89, Time 2 α = 0.85), and science cost (i.e., “I am tired after doing schoolwork on this subject,” “Studying this subject takes a lot of energy,” and “I don’t have time to do the thing I want, if I want to be good at this subject”; Time 1 α = 0.59, Time 2 α = 0.66). We used Likert-type visual scales from 1 = “Totally disagree” to 5 = “Totally agree,” in which 1 was indicated with the smallest star and 5 with the biggest star, etc.

#### Occupational Aspirations

Information on the students’ future occupational aspirations was sought using an open-ended question about their dream jobs. In the second grade, 61% of the students were able to name an occupation depicted as their dream job. The answers were classified by occupation level: support occupation (e.g., hairdresser) and professional occupation (e.g., medical doctor), with the most frequent answers being police officer, medical doctor, teacher, and professional football player. The occupations were further classified according to whether they fit in the STEM field (e.g., medical doctor, astronaut, game inventor, veterinarian) or not (e.g., sales person, football player, hairdresser, teacher). Using a coding scheme in which STEM included both physical sciences and life sciences, the answers were coded as 0 = support level and 1 = professional level and as 0 = non-STEM and 1 = STEM.

#### Background Information

Background information collected in the questionnaire included gender (0 = girl, 1 = boy) and age (i.e., date of birth).

#### Analytical Strategy

All analyses were conducted using longitudinal structural equation modeling (SEM) (Kline, 2005), as estimated by Mplus 8.0 (Muthén and Muthén, 2017). The models were estimated using the robust maximum-likelihood (MLR) estimator, which is robust against the non-normality of the observed variables and further considers the treatment of responses on a five-point Likert-type scale as the continuous variables (Beauducel and Herzberg, 2006; Hox et al., 2010; Muthén and Muthén, 2017). The MLR estimator was used in conjunction with full information maximum-likelihood (FIML) estimation in order to cope with a reasonable number of missing responses in the data. Only 30 students (9%) dropped out of the follow-up because they were absent from school on the day of the data collection due to illness or because their families had moved to another area. For similar reasons, 39 new students (12%) joined the study in the second grade. The intra-class correlations of the students’ self-concept of ability, intrinsic value, and cost were calculated at the classroom level and at the student level. The purpose was to explore whether it would be critical to analyze the model as multilevel. The class-level variances and intra-class correlations between the classes were low (ranging from 0.02 to 0.10) at both time points, indicating that the students’ motivation was mainly explained at the student level, which meant that the multilevel model was not necessary.

Model fit was evaluated by considering a wide range of descriptive goodness-of-fit indices (e.g., Marsh et al., 2004), the comparative fit index (CFI), the root mean square error of approximation (RMSEA), and the standardized root mean square residual (SRMR), which are reported with the traditional Chi-square statistics and the corresponding degrees of freedom. For the CFI and TLI, values above 0.90 and 0.95, respectively, represent an adequate and good model fit (Hu and Bentler, 1999). SRMR and RMSEA values below 0.06 and 0.08, respectively, reflect a good and acceptable fit to the data (Browne and Cudeck, 1993; Hu and Bentler, 1999).

In order to make gender comparisons, we had to ensure that the constructs were measured similarly for boys and girls and that they remained the same across time points. Thus, the group and longitudinal invariance of the factor loadings and intercepts were tested. To compare models and evaluate invariance, we examined the changes in the descriptive goodness-of-fit indices. According to the guidelines proposed by Cheung and Rensvold (2002), two models can be seen as equivalent, and invariance can be assumed as long as the change in the CFI is not more than 0.01 and the RMSEA increases by less than 0.015 for a more parsimonious model. Given the various goodness-of-fit indices and their controversial cut-off criteria for model fit evaluation, researchers are recommended to simultaneously take different goodness-of-fit indices into account and treat the respective cut-off criteria as guidelines instead of golden rules.

## Results

### Descriptive Statistics and Correlations

According to the present study’s descriptive statistics (Table 1), the students had a high science-related self-concept of ability, i.e., they felt science was interesting, and they did not perceive a high cost in science learning.

**TABLE 1:** Descriptive statistics and correlations.

	**First grade (Time 1)**									**Second grade (Time 2)**								
	
**Variables**	**1**	**2**	**3**	**4**	**5**	**6**	**7**	**8**	**9**	**10**	**11**	**12**	**13**	**14**	**15**	**16**	**17**	**18**
**Time 1**																		
1. I am good at science	−																	
2. I am good at schoolwork on this subject	0.53^∗∗^	−																
3. Schoolwork on this subject is easy for me	0.26^∗∗^	0.43^∗∗^	−															
4. I think this subject is fun	0.44^∗∗^	0.39^∗∗^	0.25^∗∗^	−														
5. I like to do schoolwork on this subject	0.51^∗∗^	0.50^∗∗^	0.19^∗∗^	0.79^∗∗^	−													
6. I just like this subject	0.39^∗∗^	0.39^∗∗^	0.26^∗∗^	0.69^∗∗^	0.69^∗∗^	−												
7. I am tired after doing schoolwork on this subject	–0.09	–0.20^∗∗^	–0.08	–0.17^∗∗^	–0.17^∗∗^	–0.17^∗∗^	−											
8. Studying this subject takes a lot of energy	–0.01	–0.08	0.00	–0.05	–0.01	–0.09	0.39^∗∗^	−										
9. I don’t have time to do the thing I want, if I want to be good in this subject	–0.02	–0.06	0.06	–0.17^∗∗^	–0.20^∗∗^	–0.16^∗∗^	0.34^∗∗^	0.24^∗∗^	−									
**Time 2**																		
10. I am good at science	0.19^∗∗^	0.12	–0.03	0.01	0.00	0.01	–0.01	–0.04	–0.01	−								
11. I am good at schoolwork on this subject	0.23^∗∗^	0.23^∗∗^	0.07	0.15^*^	0.11	0.13^*^	0.00	–0.10	–0.01	0.35^∗∗^	−							
12. Schoolwork on this subject is easy for me	0.10	0.19^∗∗^	0.24^∗∗^	0.05	0.04	0.05	–0.04	–0.05	–0.09	0.32^∗∗^	0.40^∗∗^	−						
13. I think this subject is fun	0.19^∗∗^	0.03	0.00	0.26^∗∗^	0.19^∗∗^	0.28^∗∗^	–0.09	–0.08	−0.16^*^	0.29^∗∗^	0.47^∗∗^	0.24^∗∗^	−					
14. I like to do schoolwork on this subject	0.20^∗∗^	0.16^∗∗^	0.04	0.20^∗∗^	0.21^∗∗^	0.25^∗∗^	–0.05	–0.05	–0.17^∗∗^	0.32^∗∗^	0.40^∗∗^	0.32^∗∗^	0.67^∗∗^	−				
15. I just like this subject	0.27^∗∗^	0.15^*^	0.00	0.28^∗∗^	0.29^∗∗^	0.33^∗∗^	–0.07	–0.08	–0.11	0.24^∗∗^	0.40^∗∗^	0.27^∗∗^	0.60^∗∗^	0.68^∗∗^	-			
16. I am tired after doing schoolwork on this subject	–0.18^∗∗^	−0.16^*^	−0.14^*^	−0.14^*^	−0.14^*^	–0.17^∗∗^	0.20^∗∗^	0.17^∗∗^	0.04	–0.20^∗∗^	–0.26^∗∗^	–0.20^∗∗^	–0.20^∗∗^	–0.24^∗∗^	-0.25^**	-		
17. Studying this subject takes a lot of energy	−0.15^*^	–0.23^∗∗^	−0.15^*^	−0.13^*^	−0.16^*^	−0.14^*^	0.12	0.20^∗∗^	0.01	–0.10	–0.28^∗∗^	–0.29^∗∗^	–0.20^∗∗^	–0.20^∗∗^	-0.19^**	0.58^**	-	
18. I don’t have time to do the thing I want, if I want to be good in this subject	–0.06	–0.09	0.00	–0.12	–0.09	–0.19^∗∗^	0.11	0.18^∗∗^	0.21^∗∗^	–0.16^∗∗^	–0.16^∗∗^	−0.13^*^	–0.19^∗∗^	–0.22^∗∗^	-0.14^*	0.34^**	0.25^**	-
*M*	4.06	4.19	3.88	3.78	3.79	3.71	2.7	2.64	2.58	3.82	3.96	3.86	3.83	3.84	3.87	2.64	2.39	2.27
*SD*	1.102	1.105	1.271	1.467	1.401	1.543	1.708	1.62	1.641	1.009	1.048	1.163	1.318	1.25	1.212	1.51	1.485	1.471
Range	4	4	4	4	4	4	4	4	4	4	4	4	4	4	4	4	4	4

^*^Correlation is significant at the 0.05 level (two-tailed). ^∗∗^Correlation is significant at the 0.01 level (two-tailed).

To examine the factor structure of science-related self-concept of ability, intrinsic value, and cost, confirmatory factor analysis (CFA) was employed. Correlations between the latent factors are given in Table 2 (see Supplementary Table S1, for factor loadings and effect sizes of measured items in science task value scale). The estimated latent correlations were drawn from the strong measurement model with equal intercepts. All latent correlations among self-concept, intrinsic value, and cost within the time points were statistically significant. Moreover, self-concept of ability in the first grade correlated positively with intrinsic value and cost a year later, and intrinsic value in the first grade correlated negatively with cost a year later. However, the correlations between the first graders’ science-related intrinsic value and cost and their later self-concept of ability were not significant.

**TABLE 2:** Estimated correlation matrix for the latent variables.

	**First grade (T1)**			**Second grade (T2)**					
**Variable**	1	2	3	4	5	6	7	8	9
1. Self-concept (T1)	−								
2. Intrinsic value (T1)	0.67^∗∗∗^	−							
3. Cost (T1)	–0.2^∗∗^	–0.25^∗∗∗^	−						
4. Self-concept (T2)	0.33^∗∗∗^	0.15	–0.11	−					
5. Intrinsic value (T2)	0.24^∗∗^	0.33^∗∗∗^	–0.16	0.7^∗∗∗^	−				
6. Cost (T2)	–0.32^∗∗∗^	–0.23^∗∗∗^	0.27^∗∗^	–0.49^∗∗∗^	–0.37^∗∗∗^	−			
7. Occupation level (T2)	0.17	0	–0.06	0	–0.3	−0.16^*^	−		
8. STEM (T2)	–0.02	0.16^*^	−0.18^*^	–0.06	0.04	–0.04	0.06	−	
9. Gender	0.03	–0.16^∗∗^	0.15^*^	−0.16^*^	–0.11	0.06	0.12	–0.22^∗∗∗^	–
*M g*irl	0.00	0.00	0.00	–0.14	0.04	–0.17			
*M* boy	–0.03	−0.36^*^	0.20	−0.36^*^	–0.20	0.02			
*Std.Error* girl	0.00	0.00	0.00	0.11	0.10	0.11			
*Std.Error* boy	0.14	0.14	0.14	0.14	0.13	0.14			

Latent means by gender are compared to girls’ fixed mean in T1. ^∗∗^Correlation is significant at the 0.01 level (two-tailed). ^*^Correlation is significant at the 0.05 level (two-tailed).

Three-factor measurement models were specified for both boys and girls as well as separately for the first and second graders. The fit indices of the models were considered to be good (Table 3). After satisfactory measurement models were found separately for gender and each grade, we tested the measurement invariance of the CFA models across time and gender. The configurally invariant CFA models, where no constraints were placed on any of the parameter estimates, fit the data well (Table 3). Testing for weak measurement invariance involved constraining each corresponding factor loading to be equal across gender and time, while strong measurement invariance also involved equalizing the corresponding intercepts across gender and time. The change in model fit between the configural and weak model as well as the change between the weak and strong model were modest and considered acceptable (Table 3). Since the multiple-group models were invariant, we decided to collapse the covariance information across the groups and specify the full SEM as a single-group model, while setting gender as a covariate (Little et al., 2007).

**TABLE 3:** Model fit statistics for the longitudinal confirmatory factor analysis (CFA) and structural equation modeling (SEM) models.

**Models**	***df***	χ^2^	**Scaling correction factor**	***p***	**RMSEA**	**CFI**	**TLI**	**SRMR**
**CFA models separately for boys and girls**								
CFA boy time 1	24	25.00	0.99	0.406	0.018	0.997	0.996	0.042
CFA boy time 2	24	23.58	1.14	0.486	0	1	1.003	0.043
CFA girl time 1	24	39.29	1.20	0.025	0.062	0.954	0.932	0.047
CFA girl time 2	24	19.03	1.24	0.751	0	1	1.028	0.034
Longitudinal multiple-group models	222	265.43	1.06	0.024	0.034	0.969	0.958	0.059
Configural across gender and time								
Factor loading invariance across gender and time	240	287.99	1.07	0.018	0.035	0.966	0.957	0.07
Intercept invariance across gender and time	258	312.26	1.06	0.012	0.036	0.962	0.955	0.071
**Single-group CFA models**								
CFA time 1	24	50.24	1.11	0.001	0.06	0.961	0.941	0.041
CFA time 2	24	28.07	1.24	0.257	0.024	0.992	0.988	0.031
Configural across time	111	147.45	1.11	0.012	0.031	0.974	0.964	0.043
Factor loading invariance across time	117	155.59	1.12	0.010	0.031	0.972	0.964	0.049
Intercept invariance across time	123	168.26	1.12	0.004	0.033	0.967	0.959	0.049
**SEM models**								
Cross-lagged panel model with gender as a covariate	138	185.46	1.10	0.004	0.032	0.966	0.958	0.049
CLPM with logistic regression (STEM aspirations as outcome)	162	206.59	1.09	0.010	0.029	0.97	0.961	0.048

### Results for Research Question 1: What Are the Autoregressive and Cross-Lagged Effects Between Science-Related Self-Concept of Ability, Intrinsic Value, and Cost Across the First and Second Grades?

**FIGURE 1:**
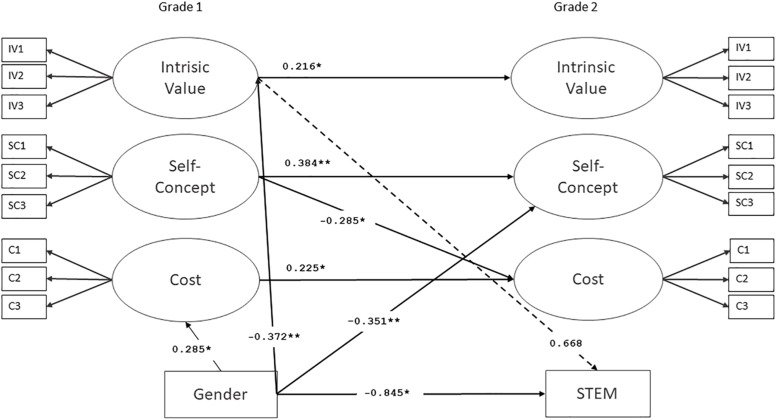
Cross-lagged panel model for students’ task motivation and occupational aspirations. IV, intrinsic value; SC, self-concept; C, cost. ^*^p < 0.015, ^∗∗^*p* < 0.01, ^∗∗∗^*p* < 0.001.

After establishing measurement invariance, we investigated the autoregressive and cross-lagged effects of self-concept of ability, intrinsic value, and cost across the first and second grades, using a cross-lagged panel model. The cross-lagged panel model is used to examine reciprocal relationships or directional influences between variables across time (Kearney, 2017). Autoregressive effects describe the stability of the construct between measurement points, while cross-lagged effects indicate the association between two variables across time. The results showed that students’ science-related self-concept of ability was somewhat stable (β = 0.384, *p* = 0.004), while there was a great deal of fluctuation in their science-related intrinsic value (β = 0.216, *p* = 0.039) and cost (β = 0.225, *p* = 0.047). Large regression coefficients indicate greater stability, while small regression coefficients indicate more variance in the construct, i.e., less stability across time (Kearney, 2017). To examine the interrelations of the students’ task values, cross-lagged effects between self-concept of ability, intrinsic value, and cost were investigated. The only significant cross-lagged effect was found between self-concept of ability and cost: A higher self-concept of ability in science in the first grade predicted lower cost in science in the second grade (Figure 1).

### Results for Research Question 2: Do First Graders’ Science-Related Task Values Predict Their Future STEM Occupational Aspirations 1 Year Later?

Students’ future STEM occupational aspirations and levels of occupational aspirations (hence, educational aspirations) were analyzed by regressing occupational aspirations on first-grade science motivation and gender. Since the variables for occupational and educational aspirations were dichotomous, logistic regression was used to model the relationship between these aspirations and science motivation. The results show that first-grade science intrinsic value was a marginally significant predictor of future STEM occupational aspirations in the second grade (β = 0.668, *p* = 0.056).

### Results for Research Question 3: Are There Gender Differences in Students’ Science-Related Task Values and Future STEM Occupational Aspirations in the First and Second Grades?

Finally, results indicate that girls were more interested in science than boys in both the first and the second grade (T1: *Z* = 0.364, *SE* = 0.141, *p* = 0.10; T2: *Z* = 0.241, *SE* = 0.118, *p* = 0.041). The mean levels of the girls’ science task values remained stable across the first and second grades, while the mean levels of the boys’ self-concept of ability decreased over the study’s 1-year period (*Z* = 0.330, *SE* = 0.128, *p* = 0.010) (Table 2). The cross-lagged model revealed that the boys were more likely to experience higher cost in science learning in the first grade, and the girls were more likely to have a higher self-concept of ability in science in the second grade while controlling for the first grade motivation variables. In addition, the girls reported more future STEM occupational aspirations than the boys, when both genders had a similar level of EVT motivation. The regression coefficients for the gender effects are presented in Figure 1.

## Discussion

Our aim in the present study was to examine the stability and interrelations of early elementary students’ self-concept of ability, intrinsic value, and cost in science learning within a 1 year time period. We also examined whether these motivational beliefs were associated with students’ occupational aspirations and whether these beliefs differed between genders at the beginning of their school careers. The study had four major findings, expanding the existing literature on young students’ science motivation. First, we found that students’ science-related self-concept of ability and intrinsic value were high and that they perceived a low cost in science learning in the first and second grades. Some stability in students’ self-concept of ability and positive and negative science motivation were found, but there was also a great deal of fluctuation in the rank-order of intrinsic value and cost across the first and second grades. Second, only one cross-lagged effect between the motivational beliefs was significant: high self-concept of ability was linked to low cost a year later. Third, the students’ high intrinsic value in science in Grade 1 marginally significantly predicted their STEM aspirations in Grade 2. Fourth, we found that compared to boys, girls had higher science motivation at both time points, and higher self-concept of ability in the second grade. Boys perceived higher cost in science learning in the first grade compared to girls. Moreover, girls reported more STEM aspirations in the second grade. These findings suggest that girls are initially motivated in science and that it is worthwhile to investigate gendered trajectories in STEM as early as possible.

### Stability and Interrelations of Science-Related Self-Concept of Ability, Intrinsic Value, and Cost Across the First and Second Grades

First, we found that the students’ self-concept of ability and intrinsic value were high and that they perceived a low cost in science learning in the first and second grades. The different science-related EVT components were already separable among the first graders, supporting earlier studies on mathematics and languages (Eccles et al., 1993; Viljaranta et al., 2016). Students, especially the girls, were motivated in science and perceived themselves as skillful at the beginning of their school careers. This result confirms our hypothesis and is in accordance with those of previous studies, which reported that at the beginning of their school careers, children are typically optimistic about their abilities (e.g., Stipek and Mac Iver, 1989) and show high levels of intrinsic value toward various school subjects (e.g., Gottfried et al., 2001; Viljaranta et al., 2016). These results vary considerably from the results usually found for students at the end of compulsory education. According to the PISA 2015 (OECD, 2016) affective measurements, Finnish students reported the fourth lowest enjoyment in science learning among OECD countries. Moreover, students’ self-concept of ability and positive and negative motivation were somewhat stable; however, there was also a great deal of fluctuation in these constructs across Grades 1 and 2. The results show that in terms of the science-related self-concept of ability, there was some within-student (rank order) stability between the time points, whereas intrinsic value and cost fluctuated. This finding is in line with our hypotheses, and confirms existing literature indicating that the development of students’ motivational beliefs is not very stable from the age of 7–8 years (Wigfield and Eccles, 1992; Eccles and Wigfield, 2001). The literature also suggests that science-related self-concept of ability, interest, and cost are still malleable at the beginning of elementary school, making it possible to influence the formation of young students’ science motivation.

Second, the present study reveals a negative link between self-concept of ability and cost after 1 year. This finding is in line with our hypothesis and the EVT (Eccles et al., 1983; Eccles and Wigfield, 2001) and suggests that science-related exhaustion might result from a lack of self-evaluated abilities. The finding gives depth to existing literature on young students’ science motivation, especially in relation to cost, which has not been studied before. As an educational implication, we need to emphasize intrinsic value while planning science lessons, and teachers need to ensure that students are able to do the science tasks. Strengthening students’ ability beliefs by providing supportive teaching practices, and emphasizing formative and encouraging assessment in the early elementary years would be crucial. In addition, by organizing interesting science activities and avoiding ranking-oriented summative assessment, we might be able to engage students in science learning and encourage them to develop STEM aspirations.

### First Graders’ Science Interest and Future STEM Occupational Aspirations

Our results showed that students’ high intrinsic value in science in first grade predicted their STEM aspirations marginally significantly in the second grade. Although the relation between science intrinsic value and future STEM occupational aspirations was only marginally significant, the effect size was rather large, suggesting a link between interest and STEM aspirations in young students. This tentative finding, which should be interpreted with caution, is in line with existing literature on students’ interest and related STEM aspirations in middle and high school (Guo et al., 2015b, 2017). The poor probability value might be due to missing data, since one-third of the students in the second grade could not name a dream job. Still, a high intrinsic value in science seems to evoke or perhaps create possibilities for imagining future occupational aspirations at the beginning of elementary education. This raises the question of whether intrinsic value in school science starts to direct students’ career choices as early as age 7. At the very least, it does seem that the links regarding science motivation and STEM aspirations emerge very early. As an educational implication, since one-third of the students did not mention their dream occupation, STEM occupations should be introduced in elementary school to increase students’ awareness (Miller et al., 2018) and to connect STEM occupations to situations where intrinsic value is emphasized in school.

### Gender Differences in Students’ Science Task Values and Future STEM Occupational Aspirations

In this study, girls had higher science motivation and self-concept of ability than boys at the beginning of elementary school. The boys perceived greater cost in their science learning than the girls, and their science-related self-concept of ability decreased from the first to the second grade. The results revealed the importance of gender differences in school science learning; the girls were more interested in science in the first grade and had a higher self-concept of ability and more future STEM occupational aspirations in the second grade than the boys. It seems that gender differences in valuing STEM start to develop early. However, gender effects in the present study partly contradict earlier findings in science interest (e.g., Leibham et al., 2013) and self-concept of ability (e.g., Guo et al., 2018b), which showed that girls have a higher science-related task motivation and self-concept of ability than boys. Previous research has also showed that although globally boys tend to present higher science self-concept than girls, in some countries the gender gap was wider and in other countries narrower (Wilkins, 2004).

Discrepancy in the findings might reflect developmental changes in students’ science motivation. It has been shown that boys have higher self-concept of ability in mathematics and higher value in mathematics learning in elementary school (Eccles et al., 1983), and that girls’ mathematics interest is significantly lower than that of boys’ in middle school (Gaspard et al., 2015; Guo et al., 2015b, 2017). The changing character of science and the increasing intensity of mathematics in physics and chemistry in middle school might influence the later decline in girls’ science motivation. It is also possible that there are some unique elements in the Finnish education system that explain the present results on gender differences in early science motivation. For example, boys’ underachievement and general lack of motivation in school, and the strong promotion of gender equality in schools, which the Pisa 2015 data highlight: Finnish girls’ are outperforming boys and the majority of the students in other OECD countries (OECD, 2016). In addition, girls might be more mature and ready for school demands than boys, who experience greater cost in science learning. However, as the Pisa 2015 results also showed, boys have more positive attitudes to science than girls at the end of compulsory education, which indicates that girls’ science interest also declines in Finland (OECD, 2016).

In the current study, girls reported more STEM aspirations than boys in Grade 2. In prior studies, boys have been found to have more STEM aspirations than girls (Eccles, 2011; Wang and Degol, 2013), but these studies were conducted among middle and high school students. It has been suggested that girls’ low science interest and lack of STEM-related career aspirations result from gender socialization (Watt et al., 2012). Prior studies show that stereotypical beliefs on gender roles are well-developed before the start of formal education influencing children’s interest (see Bian et al., 2017) although there might be an ongoing generational shift in children gender stereotypes in science (Miller et al., 2018). The media, students’ peers, parents, and teachers might promote gender stereotypes in which girls are not expected to be interested or achieve success in mathematics and science, which might prevent gender-atypical behaviors (Watt, 2016). Thus, the results of the current study might vary with age. Girls’ higher STEM aspirations in this study could be related to interest in life sciences, which in our coding were included in the STEM occupation category. Thus, those girls whose dream job was to be a medical doctor or a veterinarian were included as having occupational STEM aspirations. It is important to follow the development of science interest in these students to investigate possible changes that might occur when they transition to middle school.

## Conclusion

In conclusion, young students’ science motivation was rather high at the beginning of elementary school, but the mean levels of intrinsic value declined during the first year. Science-related self-concept was more stable compared to intrinsic value and cost in science learning, and high self-concept of ability seemed to buffer against perceived cost. Students’ high motivation was related to STEM occupational aspirations. Gendered differences in science motivation were found at the age of 7 years, and favored girls.

### Limitations and Further Research

The SEM model in the current study was not analyzed as a multilevel model, which would have been appropriate as the students were nested within classrooms and schools. However, as the class-level variances and the intra-class correlations between the classes were small, and the number of classrooms was insufficient to take the hierarchical structure adequately into account, we decided not to use the multilevel model. In future studies, it would be important to analyze data collected from students in different classrooms and schools as multilevel.

We acknowledge that predicting the occupational aspirations of students in the first years of elementary school is far-fetched, since their knowledge of possible careers is limited, influenced by their parents’ occupations and occupations visible in media, especially TV animations, being perhaps rather traditional or fictional. Unfortunately, students’ awareness of their parents’ occupations was too limited for us to use as an indicator of their socio-economic status. Now that the students are older and their awareness of their parents’ jobs might be clearer, their parents’ occupations will be the subject of further enquiry in a follow-up data collection effort. Moreover, more sensitive career coding in science (e.g., physical sciences and life sciences) is crucial to draw conclusions on possible changes in gendered pathways in STEM.

In order to understand students’ low science motivation, it would be worthwhile to investigate the development of their motivational trajectories across their elementary, middle, and high school education to try to identify *how* science motivation develops across school years, *when* changes occur, and *why* these changes occur. It would also be necessary to compare students’ views of science to their views of mathematics and language to examine more closely the relationships between school subjects in students’ motivational beliefs. In the future, we plan to investigate whether these gender effects remain unchanged or whether later on in their schooling boys develop higher science motivation levels than girls and if so, why and when. Moreover, the link between science interest and STEM aspirations in young students calls for further research into the tentative finding of the current study.

## Ethics Statement

The research project follows strict national ethical guidelines regarding scientific studies of human subjects set by the Finnish Advisory Board of Research Integrity (TENK, https://www. tenk.fi/en), which are in line with the European Code of Conduct for Research Integrity of ALLEA and the General Data Protection Regulation recently issued by the European Commission. The University of Helsinki Ethical Review Board in the Humanities and Social and Behavioural Sciences sanctions these national guidelines (TENK) and provides six descriptions of research designs that require ethical review (see https://www.helsinki.fi/sites/default/files/atoms/files/when_are_ethical_reviews_required.pdf). According to these guidelines, this study did not require ethical review, and therefore, no application was made for an ethics approval. Furthermore, to follow good scientific practice, the research plan was pre-examined and approved by the Education Division of the city of Helsinki. Since the participants of the study were elementary school-aged children, the description of the study and permission forms for participation were sent to the students’ parents beforehand. Parental consent was sought, and parents had the opportunity to decline their child’s participation in the study. Written informed parental consent was obtained for all the student participants. The data collection was integrated in the students’ normal classroom activities. The headmasters and teachers of the participating schools were informed about and agreed to the data collection schedule. The class teacher organized separate activities for the students who did not have permission to participate in the study.

## Author Contributions

JV-L and JG performed the analytic calculations. JV-L wrote the manuscript, with the help of JG and KS-A. KS-A conceived the original idea. KJ and AL collected the data. JL and KS-A helped supervise the project. All authors discussed the results and contributed to the final manuscript.

## Conflict of Interest Statement

The authors declare that the research was conducted in the absence of any commercial or financial relationships that could be construed as a potential conflict of interest.

## Footnotes

1 https://www.tenk.fi/en

2 https://www.helsinki.fi/sites/default/files/atoms/files/when_are_ethical_reviews_required.pdf (accessed May 2019).
