# Supplementary material for: The Relations of Science Task Values, Self-Concept of Ability, and STEM Aspirations Among Finnish Students From First to Second Grade
Source: Front Psychol. 2019 Jul 2;10:1449. doi: 10.3389/fpsyg.2019.01449 (PMC6614377; doi:10.3389/fpsyg.2019.01449)
Supplement: Supplementary file 1 [file Table_1.docx]

**Supplemental materials**

Table S1. Factor Loadings and Effect Sizes of Measured Items in Science Task Value Scale

|  | Time 1 | | |  | Time 2 | | |  |  |
| --- | --- | --- | --- | --- | --- | --- | --- | --- | --- |
| Items | Ability Self-concept | Intrinsic value | Cost value |  | Ability Self-concept | Intrinsic value | Cost value | R2 | Stand. Error of R2 |
| *Time 1* |  |  |  |  |  |  |  |  |  |
| 1. I am good at science | 0.66 |  |  |  |  |  |  | 0.43 | 0.07 |
| 2. I am good at the schoolwork regarding this subject | 0.81 |  |  |  |  |  |  | 0.65 | 0.08 |
| 3. The schoolwork regarding this subject is easy to me | 0.51 |  |  |  |  |  |  | 0.26 | 0.06 |
| 4. I find science fun |  | 0.87 |  |  |  |  |  | 0.75 | 0.05 |
| 5. I like to do schoolwork regarding this subject |  | 0.92 |  |  |  |  |  | 0.84 | 0.04 |
| 6. I just like the subject |  | 0.77 |  |  |  |  |  | 0.60 | 0.06 |
| 7. I’m tired after I've done schoolwork regarding the subject | |  | 0.71 |  |  |  |  | 0.50 | 0.08 |
| 8. Studying the subject takes a lot of energy |  |  | 0.59 |  |  |  |  | 0.34 | 0.06 |
| 9. I don't have the time to do the thing I want, if I want to be good in the subject |  |  | 0.41 |  |  |  |  | 0.17 | 0.05 |
| *Time 2* |  |  |  |  |  |  |  |  |  |
| 10. I am good at science |  |  |  |  | 0.57 |  |  | 0.33 | 0.05 |
| 11. I am good at the schoolwork regarding this subject |  |  |  |  | 0.71 |  |  | 0.50 | 0.08 |
| 12. The schoolwork regarding this subject is easy to me |  |  |  |  | 0.48 |  |  | 0.24 | 0.05 |
| 13. I find science fun |  |  |  |  |  | 0.78 |  | 0.61 | 0.05 |
| 14. I like to do schoolwork regarding this subject |  |  |  |  |  | 0.85 |  | 0.71 | 0.06 |
| 15. I just like the subject |  |  |  |  |  | 0.79 |  | 0.62 | 0.06 |
| 16. I am tired after I've done schoolwork regarding the subject | |  |  |  |  |  | 0.82 | 0.66 | 0.09 |
| 17. Studying the subject takes a lot of energy |  |  |  |  |  |  | 0.68 | 0.46 | 0.07 |
| 18. I don't have the time to do the thing I want, if I want to be good in the subject |  |  |  |  |  |  | 0.45 | 0.21 | 0.05 |

**Equations for model fit**

**Equation for Tucker Lewis Index (TLI)**

“A problem with the Bentler-Bonett index is that there is no penalty for adding parameters.  The Tucker-Lewis index (also called the non-normed fit index or NNFI), another incremental fit index, does have such a penalty.  Let χ^2^/df be the ratio of chi square to its [degrees of freedom](http://davidakenny.net/cm/basics.htm#Degrees), and the TLI is computed as follows:

χ^2^/df(Null Model) - χ^2^/df(Proposed Model)

_________________________________

χ^2^/df(Null Model) - 1

If the index is greater than one, it is set at one.  It is interpreted as the Bentler-Bonett index.  Note that for a given model, a lower chi square to *df* ratio (as long as it is not less than one) implies a better fitting model.   Its penalty for complexity is χ^2^/df.  That is, if the chi square to df ratio does not change, the TLI does not change.”

**Equation for Comparative Fit Index (CFI)**

“This incremental measure of is directly based on the non-centrality measure.  Let d = χ^2^ - *df* where *df* are the degrees of freedom of the model.  The Comparative Fit Index or CFI equals”

d(Null Model) - d(Proposed Model)
d(Null Model)

“If the index is greater than one, it is set at one and if less than zero, it is set to zero. It is interpreted as the previous incremental indexes.”

**Root Mean Square Error of Approximation (RMSEA)**

“This absolute measure of fit is based on the non-centrality parameter.  Its computational formula is:

√(χ^2^ - df)

__________

√[df(N - 1)]

where N the sample size and *df* the [degrees of freedom](http://davidakenny.net/cm/basics.htm#Degrees) of the model.  If χ^2^ is less than df, then the RMSEA is set to zero.  Like the TLI, its penalty for complexity is the chi square to df ratio.  The measure is positively biased (i.e., tends to be too large) and the amount of the bias depends on smallness of sample size and df, primarily the latter.”

**Standardized Root Mean Square Residual (SRMR)**

“The SRMR is an absolute measure of fit and is defined as the standardized difference between the observed correlation and the predicted correlation.  It is a positively biased measure and that bias is greater for small N and for low df studies.  Because the SRMR is an absolute measure of fit, a value of zero indicates perfect fit.  The SRMR has no penalty for model complexity.  A value less than .08 is generally considered a good fit (Hu & Bentler, 1999).”
